# Prediction models for sepsis-associated acute kidney injury: a systematic review and meta-analysis

**DOI:** 10.3389/fendo.2026.1854549

**Published:** 2026-09-14

**Authors:** Muze Huang, Shiyuan Wu, Zi-Han Shen, Sarena Jiayao Zhang, Yaw-syan Fu, Jingyi Wu

**Affiliations:** 1 School of Medicine, Xiamen University, Xiamen, Fujian, China; 2 Center of Clinical Laboratory, Zhongshan Hospital Xiamen University, School of Medicine Xiamen, Xiamen, Fujian, China; 3 Xiamen University, Xiamen, China; and Institute of Infectious Disease, School of Medicine, Xiamen University, Xiamen, Fujian, China; 4 School of Basic Medicine, Fujian Medical University, Fuzhou, Fujian, China; 5 School of Stomatology, Xiamen Medical College, Xiamen, Fujian, China; 6 School of Clinical Medicine, Xiamen Medical College, Xiamen, Fujian, China; 7 International Institute for Integrative Sleep Medicine (WPI-IIIS), University of Tsukuba, Tsukuba, Ibaraki, Japan; 8 Department of Anatomy, School of Basic Medical Science, Xiamen Medical College, Xiamen, Fujian, China

**Keywords:** machine learning, meta-analysis, nomogram, prediction model, SA-AKI, sepsis-associated acute kidney injury, systematic review

## Abstract

**Objectives:**

This study aims to systematically evaluate the predictive performance of risk models for sepsis-associated acute kidney injury (SA-AKI). Furthermore, we explore the specific factors that influence how effectively these models perform in clinical settings.

**Methods:**

We systematically searched PubMed, The Cochrane Library, Web of Science, and Embase to identify cohort studies published up to 30 March 2026. These studies focused on the development and validation of SA-AKI prediction models. To ensure the quality of the evidence, the risk of bias was assessed using the Prediction model study Risk Of Bias ASsessment Tool (PROBAST). Data synthesis involved a random-effects model, which we used to pool C-statistics and their 95% confidence intervals (CIs). Finally, sources of heterogeneity were explored through subgroup analysis.

**Results:**

A total of 15 studies involving 46,490 patients were included in this meta-analysis. The pooled C-statistic was 0.817 (95% CI: 0.781–0.847), indicating a moderate-to-good discriminative ability for SA-AKI. However, substantial heterogeneity was observed (*I*^2^ = 92.8%, *τ*^2^ = 0.143). Subgroup analyses further elucidated the drivers of this variation. We found that while models developed in Asian regions showed a higher pooled C-statistic than those from North America (0.845 vs. 0.777), this difference was not statistically significant (*P* for interaction = 0.076). Similarly, studies with a low risk of bias yielded superior predictive performance compared to those at high risk (0.847 vs. 0.762; *P* for interaction = 0.010). Notably, model performance was not significantly influenced by the type of validation (internal vs. external) or the language of publication based on the interaction test. However, the external validation subgroup displayed an exceptionally wide 95% CI, underscoring a high degree of uncertainty in this pooled estimate due to the limited number of contributing studies. Sensitivity analysis using the leave-one-out method demonstrated the robustness of the pooled estimate, as the omitted results remained stable within a narrow range (0.805–0.826). Finally, visual inspection of the funnel plot and Egger’s test suggested no potential publication bias (*P* = 0.1891).

**Conclusion:**

Current prediction models for SA-AKI demonstrate moderate-to-good predictive performance; however, high heterogeneity was observed across the included studies. Future research should prioritize external validation and emphasize the reduction of bias risk. Furthermore, we recommend that investigators explore robust, population-specific models to enhance clinical utility and generalizability.

**Systematic review registration:**

https://www.crd.york.ac.uk/prospero/, identifier CRD420261347810.

## Introduction

Sepsis stands as a primary cause of mortality among patients in the intensive care unit (ICU), with more than 49 million new cases and approximately 11 million deaths reported globally each year (1, 2). Within this critical syndrome, acute kidney injury (AKI) is recognized as one of the most frequent and severe manifestations of organ dysfunction; it affects 40% to 50% of septic patients, which not only elevates mortality risk and hospital duration but also places a staggering financial strain on healthcare infrastructures (3–5). Although the Kidney Disease: Improving Global Outcomes (KDIGO) guidelines provide established diagnostic criteria, the underlying pathophysiology of sepsis-associated acute kidney injury (SA-AKI) remains notoriously complex, driven by an intricate interplay of inflammation, microcirculatory failure, mitochondrial impairment, and apoptosis (6–8). This complexity renders the early identification of high-risk patients a significant clinical hurdle. Since advanced stage or prolonged SA-AKI can lead to persistent renal impairment or progress to chronic kidney disease, the early screening of vulnerable individuals and the prompt implementation of renoprotective interventions are deemed essential for optimizing patient prognosis (9, 10).

Moreover, the global burden of SA-AKI extends beyond individual patient outcomes, contributing substantially to prolonged intensive care requirements, increased healthcare resource utilization, and long-term sequelae such as chronic kidney disease and reduced quality of life across diverse populations and healthcare settings (11–13). These challenges are further compounded by marked variations in incidence and severity influenced by patient demographics, comorbidities, and regional differences in sepsis management practices, underscoring the limitations of relying solely on conventional clinical parameters and static scoring systems for timely risk stratification (14, 15). Consequently, there is a growing consensus on the need for advanced, multidimensional risk prediction approaches that integrate readily available clinical data to facilitate earlier and more precise preventive strategies, ultimately aiming to mitigate the substantial morbidity, mortality, and economic impact associated with this common yet devastating complication of sepsis (16).

The development of predictive models has furnished clinical practice with essential tools for the early risk identification of SA-AKI. In recent years, the widespread adoption of electronic health records and advancements in machine learning algorithms have triggered a surge in specialized predictive models (17–20). These tools integrate multidimensional predictors—ranging from demographic characteristics and vital signs to laboratory metrics and biomarkers—to achieve individualized forecasting through quantified risk scores. For instance, Fan et al. developed a machine learning model using the Medical Information Mart for Intensive Care III (MIMIC-III) database (21); their study included over 15,000 patients and demonstrated robust discriminative power. Similarly, an antithrombin III-related nomogram was constructed by Xie et al., achieving an impressive concordance statistic (C-statistic) of 0.969 in their validation cohort (22). To enhance generalizability, Su et al. and Zhao et al. conducted multicenter external validations (23, 24). Innovation also extends to real-time systems, such as the one developed by Peng et al. using time-series clinical data, while the performance of diverse machine learning algorithms was compared by Shi et al. and Yue et al. (25–27) Collectively, these studies explore the feasibility of SA-AKI prediction from various perspectives, employing modeling techniques like traditional logistic regression, random forests, support vector machines, and deep learning across multiple validation designs.

However, despite the proliferation of existing research, significant heterogeneity persists in sample sizes, predictor selection, modeling techniques, and validation strategies. Such inconsistencies have led to widely divergent reports of model performance, with C-statistics fluctuating between 0.611 and 0.969, thereby complicating assessments of clinical utility. More importantly, a systematic integration and quantitative comparison of these predictive models is currently lacking. This gap makes it difficult for clinicians and researchers to determine which models offer optimal performance across varying scenarios or to identify the critical factors governing model efficacy. Consequently, we employed systematic review and meta-analysis methodologies to comprehensively retrieve and rigorously screen literature relevant to SA-AKI prediction models. Our objective was to quantitatively pool the discriminative indices of these models while exploring the influence of geographic region, validation type, risk of bias, and publication language. This study is designed not only to clarify the overall quality of evidence for existing models but also to provide an evidence-based foundation for the development of more robust and generalizable SA-AKI prediction tools. Ultimately, this work serves to facilitate early identification and precision intervention for septic patients, carrying substantial clinical significance and public health value.

## Methods

This study was designed following the PRISMA 2020 guidelines, and the protocol was preregistered in the PROSPERO database to ensure transparency and reproducibility. To ensure transparency and reproducibility throughout the investigative process, the research protocol was prospectively registered in the PROSPERO database (International Prospective Register of Systematic Reviews) on 22 March 2026 (Registration ID: CRD420261347810), prior to the initiation of formal literature full-text screening and data extraction. During the subsequent screening phase, minor amendments were made to the protocol’s prespecified “intervention” and “study design” fields to better align with the precise characteristics of the retrieved cohort studies. These updates were formally recorded as log changes in the PROSPERO database to ensure methodological transparency, and all final analyses were conducted in strict accordance with this modified protocol.

## Literature search strategy

A comprehensive search strategy was developed independently by two researchers and cross-checked to ensure its rigor. We searched four primary electronic databases—PubMed, The Cochrane Library, Web of Science, and Embase—from their inception to 30 March 2026. To avoid geographic and publication biases, no language or publication type filters were applied during the initial database search stage.

To maximize retrieval efficiency, we combined Medical Subject Headings (MeSH) and free-text terms. The search strings covered core concepts such as “sepsis,” “septic shock,” “acute kidney injury,” “prediction model,” “risk score,” “machine learning,” and “nomogram,” as well as “development” and “validation.” The detailed, reproducible search strategies for each database are provided in **Supplementary Table 1**. While specific search queries were tailored to the unique characteristics of each database, we also manually screened the reference lists of included studies. This supplementary approach was implemented to minimize the risk of omitting relevant literature.

The selection process was conducted independently by two researchers, who first utilized the EndNote software to remove duplicate records and then performed a preliminary screening based on titles and abstracts. We subsequently retrieved full-text articles for a secondary review, with any disagreements resolved through third-party arbitration. Although no language restrictions were enforced during the database queries, applying our strict clinical and methodological eligibility criteria during subsequent screening phases resulted in a final pool of studies published exclusively in English and Chinese.

Inclusion criteria were strictly developed using the Participants, Intervention, Comparison, Outcome, and Study (PICOS) design framework (**Table 1**), targeting adult patients (aged ≥ 18 years) with sepsis or septic shock. These studies focused on risk prediction models for SA-AKI, including both traditional statistical models and machine learning algorithms, although no standardized control group was required. We defined the primary outcome as model discrimination, which is typically expressed as the C-statistic or the area under the receiver operating characteristic curve (AUC-ROC). Both development and validation studies, utilizing retrospective or prospective cohort designs, were considered eligible for inclusion. Finally, we excluded conference abstracts, reviews, case reports, animal experiments, and studies with unvalidated models or missing C-statistics.

**Table 1:** The PICOS strategy.

PICOS	Description
Participants	Adults (≥18 years) diagnosed with sepsis or septic shock, hospitalized, used for the development or validation of sepsis-associated acute kidney injury (SA-AKI) prediction models.
Intervention	Prediction models for the risk of SA-AKI in sepsis patients.
Comparison	No unified comparator group; the main evaluation is the model's own performance.
Outcome	Model discrimination ability (AUC).
Study design	Studies involving prediction model development and validation, including both retrospective and prospective cohort studies.

The PICOS framework defining the eligibility criteria for the included studies. P, participants; I, intervention; C, comparison; O, outcome; S, study design; SA-AKI, sepsis-associated acute kidney injury; AUC, area under the receiver operating characteristic curve.

Models were defined as “unvalidated” and strictly excluded only if they were fitted and evaluated solely on the development dataset without any subsequent internal or external validation methods to correct for optimism bias. Conversely, any model that applied an appropriate validation technique was deemed to have an “acceptable validation” and was included. Following the TRIPOD (Transparent Reporting of a multivariable prediction model for Individual Prognosis or Diagnosis) statement, validation strategies were systematically categorized into two distinct levels: (1) internal validation, which assesses model performance and over-fitting within the source development population, encompassing split-sample validation (randomly partitioning a single cohort), cross-validation (e.g., k-fold techniques), bootstrapping (repeated resampling with replacement), and temporal validation (evaluation in a subsequent time frame within the same institution); and (2) true external validation, which rigorously tests the model’s generalizability in geographically or institutionally independent populations, such as completely separate medical centers, cross-regional clinical cohorts, or independent public databases (e.g., using a distinct MIMIC or Xiangya cohort separate from the development population). Notably, validation datasets collected during the same time frame but derived from entirely independent external clinical institutions were strictly categorized as true external validation due to their institutional and population independence.

### Data extraction

Standardized forms were employed by two independent researchers to facilitate the data extraction process. The collected information encompassed several key elements: the first author, publication year, study region, sample size (for both training and validation sets), the type of predictive model, and the validation methodology, alongside the C-statistic and its 95% confidence interval (CI). We contacted the original authors via email to retrieve any missing data; however, if these details remained unavailable, the omissions were documented to analyze their potential impact on the study findings. Ultimately, no additional unpublished data or missing information was obtained or incorporated into the final analysis; this systematic review refers strictly to peer-reviewed published data.

### Quality assessment of literature

The risk of bias in the included studies was evaluated using the Prediction model study Risk Of Bias ASsessment Tool (PROBAST), a specialized instrument designed specifically for predictive model research. This tool assesses four distinct domains—participants, predictors, outcomes, and analysis—assigning each a rating of low risk, unclear risk, or high risk of bias to determine the overall quality of the study. Two independent researchers performed the evaluation process, and we resolved any discrepancies through consensus or third-party arbitration. To provide a clear visualization of the findings, the PROBAST results are presented as traffic light plots and summary plots. These figures illustrate the risk of bias judgments for individual studies across all domains alongside the aggregate distribution of bias risk proportions.

### Statistical analysis

Statistical analyses were performed using the *meta* and *metafor* packages in R software (version 4.3.1). Given the expected variation across studies, we utilized a random-effects model for all meta-analyses, estimating the heterogeneity variance via the DerSimonian–Laird (DL) method. The primary analytical metric involved the logit transformation of the C-statistic, with pooled results presented as the C-statistic alongside its 95% CI. To reflect the distribution range of the true effect sizes, we also calculated 95% prediction intervals. Heterogeneity was assessed using the *I*^2^ statistic and Cochran’s *Q* test, where an *I*^2^ >50% or *P* < 0.10 indicated the presence of significant heterogeneity.

1. When the 95% CI [LL, UL] was provided, the SE in the raw scale was derived using the standard formula:


$$SE_{raw}=\frac{UL-LL}{2\times 1.96}$$

2. Alternatively, if a study lacked a reported 95% CI but provided the total sample size alongside the exact event counts− (cases, *n*_1_) and non-events (controls, *n*_2_), the SE was mathematically estimated using the classic Hanley–McNeil formula:


$$\text{SE}\left(\text{AUC}\right)\;\sqrt{\frac{\text{AUC}\left(1-\text{AUC}\right)\;+\;\left(n_{1}-1\right)\;\left(P_{1}-{\text{AUC}}^{2}\right)\;+\;\left(n_{2}-1\right)\left(P_{2}-{\text{AUC}}^{2}\right)}{n_{1}n_{2}}}$$

$$P_{1}=\frac{\text{AUC}}{2-\text{AUC}}\;P_{2}=\frac{2{\text{AUC}}^{2}}{1+\text{AUC}}$$

3. Because the excluded external validation data set lacked the sample size, event counts, and 95% CI simultaneously, it was mathematically impossible to compute its SE through either approach, necessitating its exclusion. We have corrected our original misstatement to ensure absolute accuracy.

To evaluate the robustness of our pooled estimates under substantial heterogeneity, we performed an additional sensitivity analysis. We employed the restricted maximum likelihood (REML) method for study-to-study variance (*τ*^2^) estimation combined with the Hartung–Knapp (HK) adjustment, providing a more conservative and robust framework for our random-effects model.

### Publication bias assessment

Publication bias was visually assessed using a funnel plot, which plotted the logit-transformed C-statistic on the *x*-axis against the inverse of the standard error on the *y*-axis. A vertical dashed line represented the pooled effect size, and red dashed curves delineated the 95% CI boundaries. To provide additional context, individual studies were color-coded based on their overall PROBAST risk of bias ratings. Furthermore, we conducted Egger’s linear regression test for quantitative detection, where a *P*-value of less than 0.10 was considered indicative of potential publication bias.

### Forest plot construction

The forest plot was generated to visualize the C-statistic and 95% CI for each included study. Individual effect estimates are represented by squares, while horizontal lines delineate the respective CIs; notably, the size of each square is proportional to the study’s weight in the meta-analysis. To maintain consistency with our quality assessment, these elements were color-coded according to the PROBAST risk of bias (blue for low risk, gray for unclear, and red for high risk). A diamond is positioned at the bottom of the plot to indicate the pooled estimate and its 95% CI derived from the random-effects model. Furthermore, a gray-shaded area was included to represent the 95% prediction interval, with heterogeneity metrics such as *I*^2^ and τ² clearly annotated.

### Subgroup analysis

To explore sources of heterogeneity and evaluate the stability of model performance, we conducted four prespecified subgroup analyses: by study region (North America vs. Asia), validation type (internal vs. external), publication language (English vs. Chinese), and overall PROBAST risk of bias (low vs. high). We conducted four prespecified subgroup analyses (by study region, validation type, publication language, and overall PROBAST risk of bias), which are presented via forest plots illustrating the pooled C-statistics and their corresponding 95% CI. We assessed differences between subgroups using a test for interaction, where a *P*-value of less than 0.05 was considered statistically significant.

### Sensitivity analysis

A sensitivity analysis was performed using the leave-one-out method, in which the pooled C-statistic was recalculated after sequentially excluding each study to evaluate the robustness of the meta-analysis results. These findings are presented in a forest plot that displays the pooled effect size and its 95% CI following the removal of each individual study. To determine the stability of our findings, we compared these recalculated values against the overall pooled effect size of 0.817.

## Results

A total of 1,019 records were identified through our initial database search, leaving 838 unique citations for title and abstract screening after removing duplicates. We ultimately included 15 studies for qualitative synthesis and quantitative meta-analysis, the characteristics of which are summarized in **Table 2**. The literature screening process is illustrated in detail within the PRISMA flow diagram (**Figure 1**).

**Table 2:** Data extraction results from the included studies.

No.	Author	Year	Region	Participants	Sepsis definition	SA-AKI definition	AKI time window	Clinical setting	Prediction horizon	Validation method	Candidate predictors	Final predictors	Handling of missing data	Clinical utility	Calibration
1	Fan et al. (21)	2020	N. America	Training 11,008 / Internal Validation 4,718	Sepsis 3.0	KDIGO	During ICU stay	ICU (MIMIC-III)	Early phase post-ICU admission	Internal cross-validation	42 clinical & lab variables	Diabetes mellitus, CKD, CHF, chronic liver disease, hyperbicarbonemia, lactate, BUN, etc.	Multiple Imputation	Not reported	Reported (Calibration curve)
2	Ma et al. (28)	2021	Asia	Training 232 / Internal Validation 126	Sepsis 3.0	KDIGO	Within 48h of ICU admission	Mixed ICU	Within 48h of ICU admission	Chronological validation	18 biomarkers & clinical indices	sCysC, uNAG, APACHE II, MAP	Excluded cases with missing data > 5%	Reported (DCA)	Reported (Calibration curve & H-L test)
3	Yang et al. (29)	2021	N. America	Training 2,012 / Internal Validation 859	Sepsis 3.0	KDIGO	During ICU stay	ICU (MIMIC-III)	Within 24h of ICU admission	Random split validation	35 routine monitoring metrics	LOS in ICU, baseline SCr, glucose, anemia, vasoactive drugs	Median/Mean imputation	Reported (DCA)	Reported (Calibration curve)
4	Xie et al. (22)	2021	Asia	Training 251 / External Validation 159	Sepsis 3.0	KDIGO	Within 7 days of ICU admission	ICU	Within 48h of ICU admission	Internal validation only	15 coagulation & renal indices	Male sex, cardiovascular disease, low AT-III levels	Complete case analysis	Not reported	Not reported
5	Xin et al. (30)	2022	Asia	Training 787 / Internal Validation 264	Sepsis 3.0	KDIGO	Within 7 days of ICU admission	Surgical/ICU	Within 24-48h of admission	Random split validation	24 inflammatory & coagulation indices	IL-6, fibrinogen, D-dimer, platelet count, lactate	Mean imputation for variables with <10% missingness	Reported (DCA)	Reported (Calibration curve)
6	Su et al. (23)	2024	Asia	Training 733 / External Validation 336	Sepsis 3.0	KDIGO	Within 48h of ICU admission	Multicenter ICU	Within 48h of ICU admission	Multicenter external validation	31 clinical & biomakers	Age, lactate, procalcitonin (PCT), D-dimer, APACHE II	MICE (Multiple Imputation by Chained Equations)	Reported (DCA)	Reported (Calibration curve & H-L test)
7	Zhao et al. (24)	2024	Asia	Training 198 / External Validation 77	Sepsis 3.0	KDIGO	During ICU stay	General ICU	Within 24h of ICU admission	Random split validation	28 routine clinical metrics	Respiratory rate, mechanical ventilation, platelet count, BUN, lactate	Excluded cases with severe missingness	Reported (DCA)	Reported (Calibration curve)
8	Lai et al. (31)	2024	Asia	Training 435 / Internal Validation 180	Sepsis 3.0	KDIGO	Early phase post-ICU admission	ICU	Within 24-48h of ICU admission	Random split + LASSO regression	38 molecular & clinical factors	Top 5 SHAP values: Lactate, SOFA score, PCT, D-dimer, BUN	Multiple Imputation	Reported (SHAP & DCA)	Reported (Calibration curve)
9	Lin et al. (32)	2024	Asia	Training 294 / Internal Validation 97	Sepsis 3.0	KDIGO	Within 48h of ED admission	Emergency Department (ED)	Within 48h of ED admission	Random split validation	22 emergency routine metrics	Age, vasopressor use, platelets, PCT, D-dimer	Mean/Median imputation	Reported (DCA)	Reported (Calibration curve)
10	Peng et al. (25)	2023	Asia	Training 4,764 / External Validation 1,800	Sepsis 3.0	KDIGO	During ICU stay	Multicenter ICU	Within 48h before ICU admission	Dual-center external validation	45 dynamic ICU metrics	Fluid input_day1, fluid input_day2, platelet_min_day5, length of ICU stay, hospital stay, Bun_max_day1, mechanical ventilation time	MissForest (Random forest imputation)	Reported (Clinical gain curve)	Reported (Calibration curve)
11	Shi et al. (26)	2024	N. America	Training 8,460 / Internal Validation 2,115	Sepsis 3.0	KDIGO	During ICU stay	ICU (MIMIC-IV)	Within 24h of ICU admission	Random split validation	56 ML candidate features	12 core features (including heart rate, creatinine, anion gap, etc.)	KNN Imputation (K-Nearest Neighbors)	Reported (CIC)	Reported (Brier score & Calibration curve)
12	Yue et al. (27)	2022	N. America	Training 3,176	Sepsis 3.0	KDIGO	During ICU stay	ICU (MIMIC-III)	Within 24-48h of ICU admission	10-fold CV + Independent test	64 all-dimensional features	15 features selected by Boruta (including urine output, GCS, SOFA, etc.)	Multiple Imputation (MICE)	Reported (DCA)	Reported (Calibration curve)
13	Deng et al. (33)	2020	N. America	Training 2,042 / Internal Validation 875	Sepsis 3.0	KDIGO	Within 24h of ICU admission	ICU (MIMIC-III)	Within 24h of ICU admission	Random split validation	29 baseline metrics	Age, SOFA score, BUN, lactate, MAP	Excluded indicators with > 20% missingness	Reported (DCA)	Reported (Good calibration)
14	Li et al. (34)	2024	Asia	Training 245	Sepsis 3.0	KDIGO	During ICU stay	ICU	Within 24h of ICU admission	5-fold CV + Independent set	26 clinical features	Age, lactate, SOFA score, PCT, vasoactive drugs	Complete case analysis	Reported (Online dynamic DCA)	Reported (Calibration curve)
15	Zhang et al. (35)	2024	Asia	Training 172 / Internal Validation 75	Sepsis 3.0	ADQI 28	Within 7 days of sepsis diagnosis	ICU	Within 24h of ICU admission	Random split validation	34 therapeutic metrics	SOFA score, platelets, lactate, PCT, APTT	Median imputation for variables with < 5% missingness	Reported (DCA)	Reported (Calibration curve)

Characteristics of the 15 included studies on sepsis-associated acute kidney injury prediction models. ICU, intensive care unit; MIMIC-III, Medical Information Mart for Intensive Care III; sCysC, serum cystatin C; uNAG, urinary N-acetyl-β-D-glucosaminidase.

**Figure 1:**
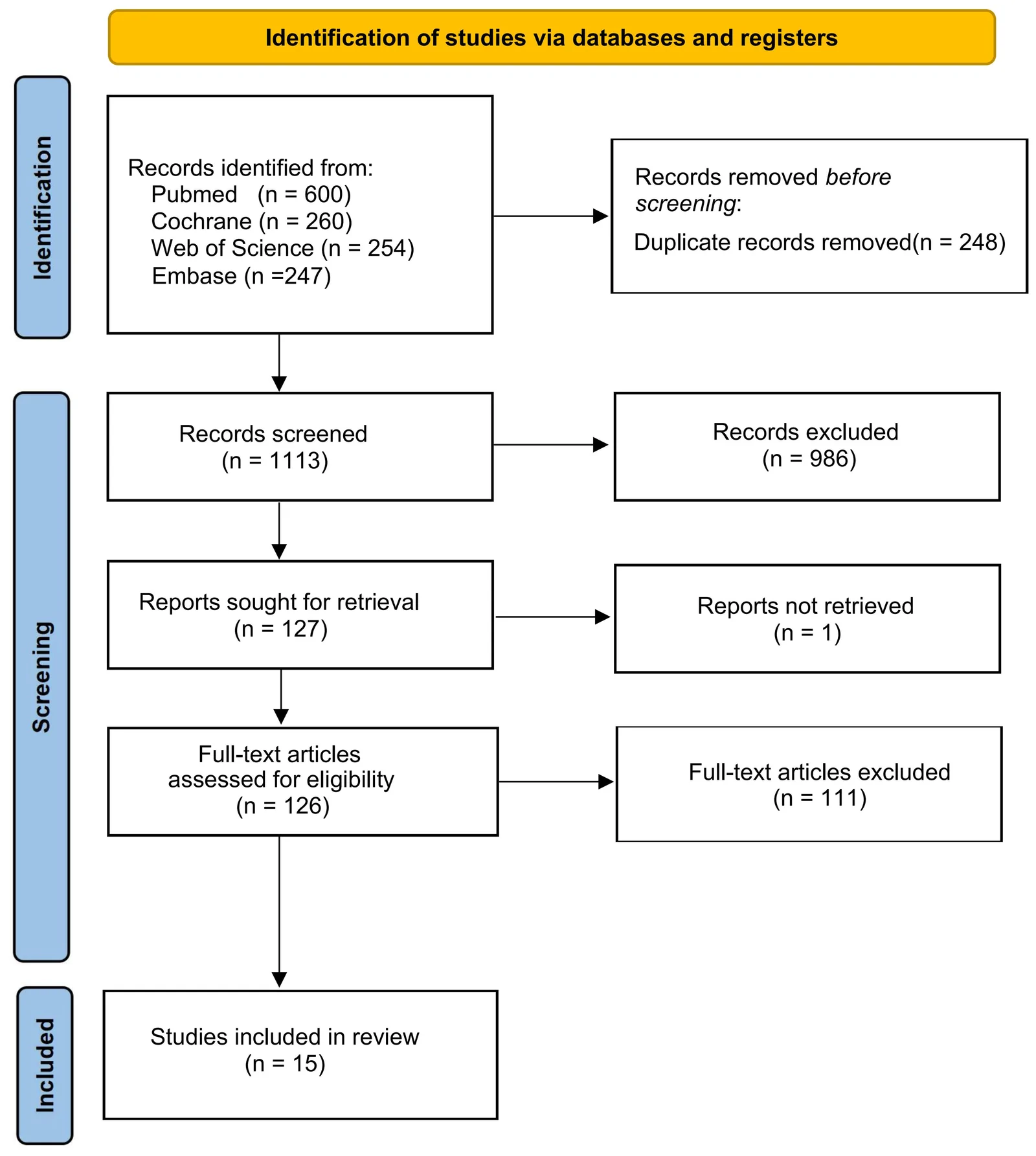
PRISMA flow diagram. The flow diagram of the study selection process according to the Preferred Reporting Items for Systematic Reviews and Meta-Analyses (PRISMA) 2020 guidelines. Records were identified from four electronic databases (PubMed, Cochrane Library, Web of Science, and Embase), and duplicates were removed using the EndNote software. Titles and abstracts were screened for eligibility, followed by full-text assessment. The final number of studies included in the qualitative synthesis and quantitative meta-analysis is indicated.

### Study characteristics

The 15 included studies were published between 2020 and 2024, with 5 conducted in North America and 10 in Asia. Sample sizes ranged significantly from 172 to 15,726 cases, while the median sample size for the training sets was calculated to be 733. Regarding validation methods, 12 studies utilized internal validation. Although four studies reported external validation cohorts (Su et al., 2024, Zhao et al., 2024, Peng et al., 2023, and Xie et al., 2021), the external validation data from Xie et al. (2021) could not be included in the subgroup meta-analysis due to missing information on the number of events, which prevented calculation of the standard error of the C-statistic. Thus, only three studies contributed to the external validation subgroup. The types of predictive models spanned a wide array of techniques, including traditional logistic regression nomograms and various machine learning algorithms, which are summarized in **Table 2**.

### Quality assessment and risk of bias

The PROBAST risk of bias assessment results are illustrated in **Figure 2**, revealing that all 15 studies (100%) were rated as low risk in the participants domain. Detailed domain-specific PROBAST assessments, including risk-of-bias judgments and supporting rationales for each of the 15 included studies, are presented in **Supplementary Table 2**. In the predictors domain, we identified 14 studies (93.3%) as low risk, while 1 study (6.7%) was categorized as unclear. Regarding the outcome domain, 10 studies (66.7%) exhibited low risk, 1 study (6.7%) was unclear, and 4 studies (26.7%) were flagged as high risk. The analysis domain showed that 14 studies (93.3%) were low risk, although 1 study (6.7%) was judged to have a high risk of bias. Overall, 9 studies (60.0%) were evaluated as having a low risk of bias, whereas 1 study (6.7%) was unclear and 5 studies (33.3%) were rated as high risk.

**Figure 2:**
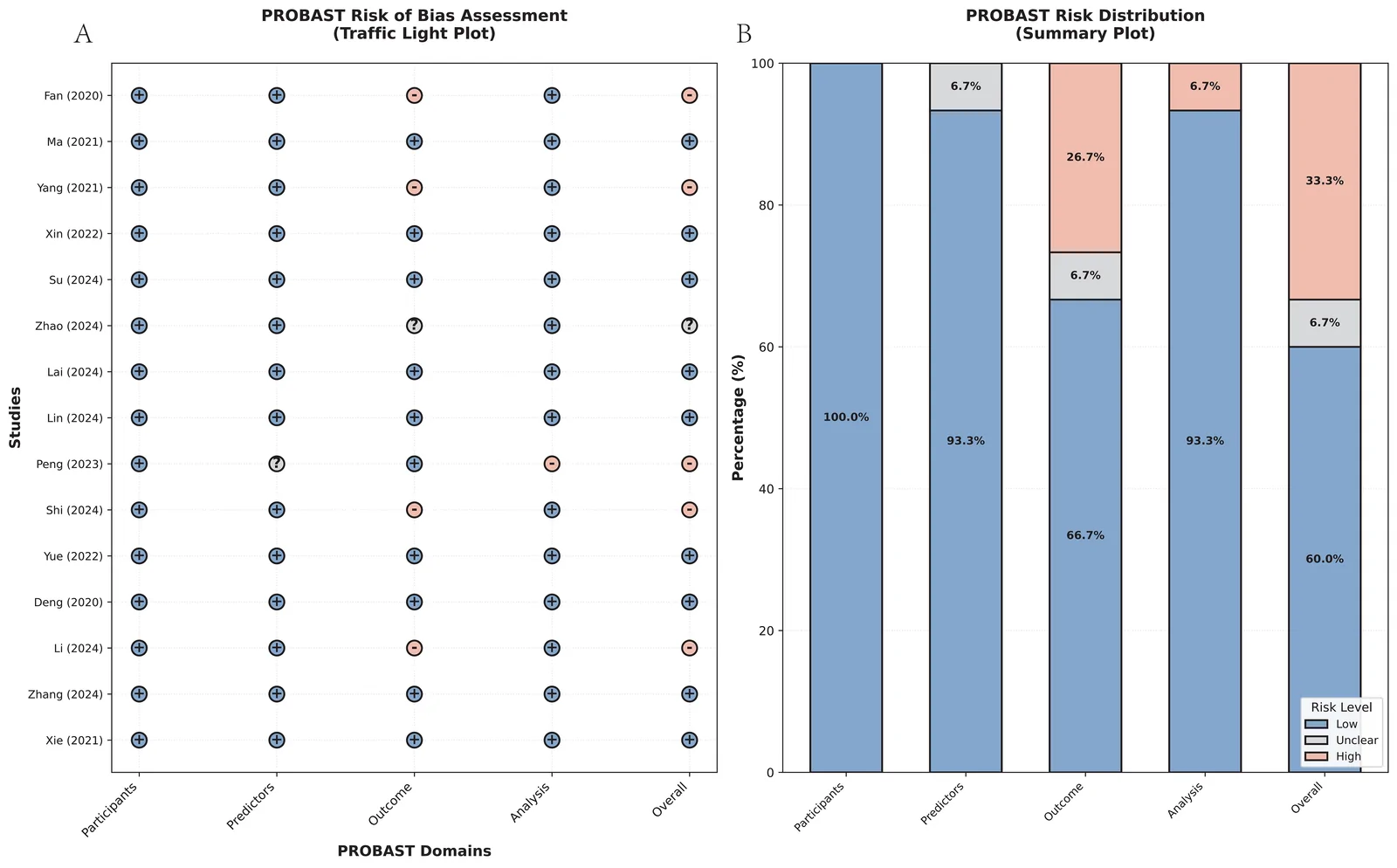
PROBAST risk of bias assessment. Risk of bias assessment using the Prediction model study Risk Of Bias ASsessment Tool (PROBAST). **(A)** Traffic light plot showing the risk of bias judgment for each of the four domains (participants, predictors, outcome, and analysis) across all 15 included studies. **(B)** Summary plot presenting the percentage distribution of low (blue), unclear (gray), and high (red) risk of bias ratings for each PROBAST domain and overall.

### Publication bias and overall predictive performance

Evaluation of the funnel plot (**Figure 3**) revealed a relatively symmetrical distribution of the included studies, suggesting no obvious publication bias. This observation was further supported by Egger’s linear regression test, which confirmed the absence of statistically significant publication bias (*P* = 0.1891). Regarding the overall predictive performance of the SA-AKI models, our meta-analysis results (**Figure 4**) yielded a pooled C-statistic of 0.817 (95% CI: 0.781–0.847), indicating that these models possess moderate-to-good discriminatory power. However, significant heterogeneity was observed across the included studies (*I*^2^ = 92.8%, *τ*² = 0.143, *P* < 0.001), and the 95% prediction interval ranged from 0.655 to 0.915, reflecting a broad distribution of the true effect sizes. The C-statistics of individual studies fluctuated between 0.611 and 0.969; specifically, the study by Xie et al. reported the highest C-statistic (0.969), while the lowest (0.611) was observed in the study by Peng et al.

**Figure 3:**
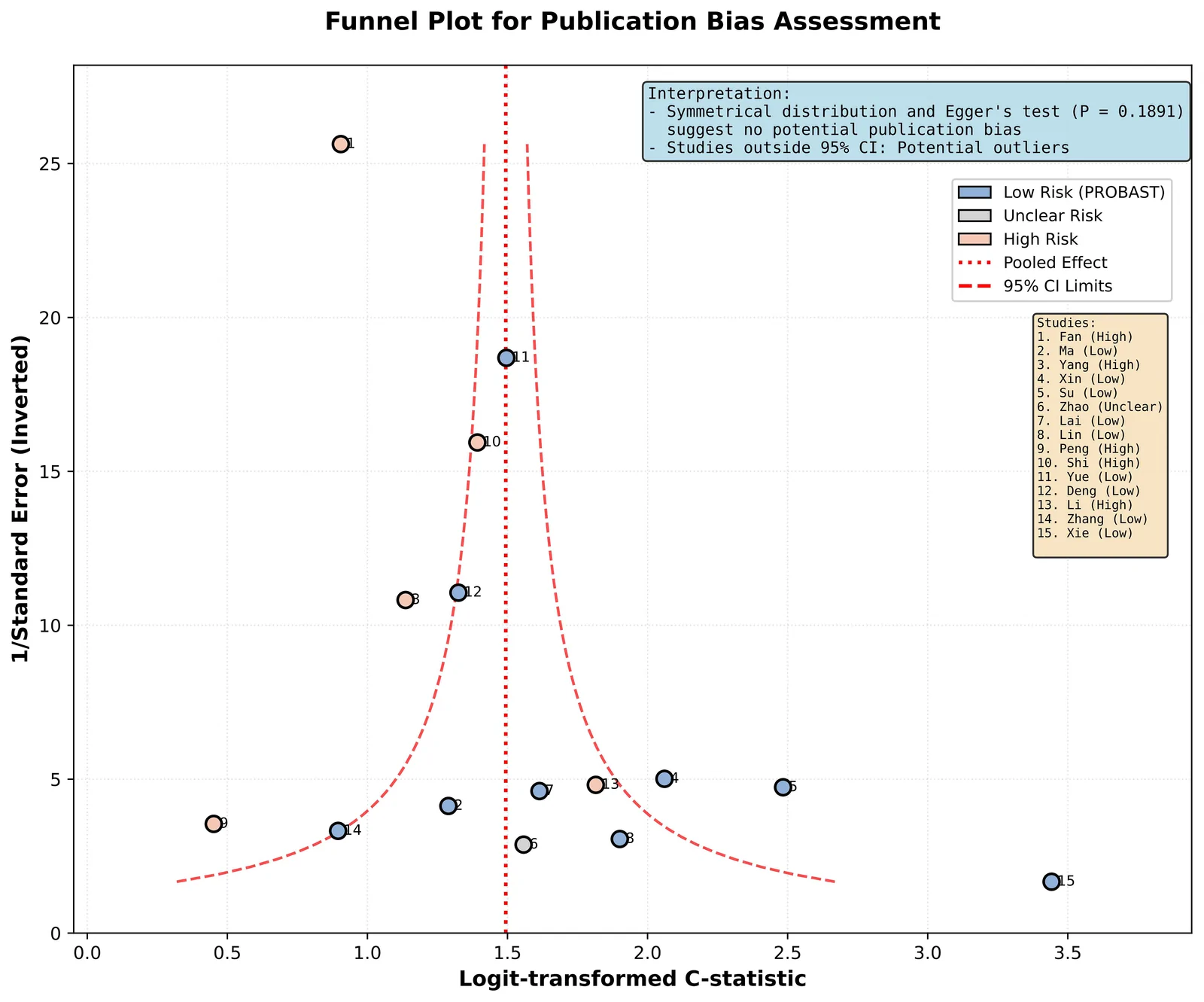
Funnel plot for publication bias assessment. Funnel plot assessing potential publication bias. The *x*-axis represents the logit-transformed C-statistic (discrimination performance), and the *y*-axis represents the inverse of the standard error (precision). The vertical dotted red line indicates the pooled effect estimate (0.817), and the dashed red curves represent the 95% CI limits. Studies are color-coded by overall PROBAST risk of bias: blue for low risk, gray for unclear risk, and red for high risk.

**Figure 4:**
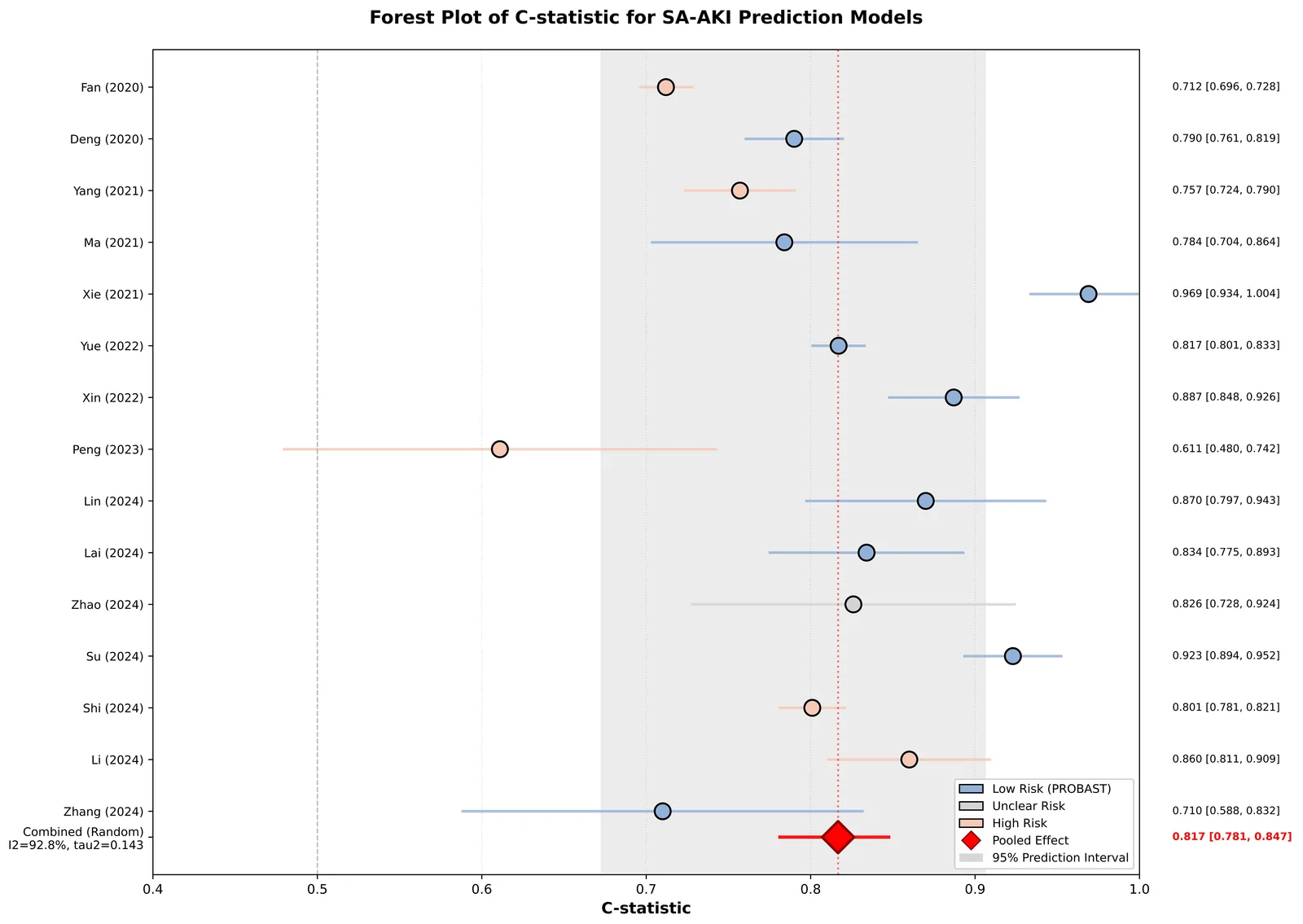
Forest plot of C-statistic for SA-AKI prediction models. Forest plot showing the C-statistic (area under the receiver operating characteristic curve) for sepsis-associated acute kidney injury prediction models across 15 included studies. Each study is represented by a square (effect estimate) and horizontal line (95% CI), with the size of the square proportional to the study weight. Studies are color-coded by overall PROBAST risk of bias: blue for low risk, gray for unclear risk, and red for high risk. The diamond at the bottom represents the pooled random-effects estimate with its 95% CI. The gray-shaded area indicates the 95% prediction interval. Heterogeneity statistics: *I*^2^ = 92.8%, *τ*² = 0.143.

### Subgroup analysis

The results of the subgroup analysis revealed several critical factors influencing model performance (**Figure 5****).** When stratified by study region, the pooled C-statistic for North American studies was 0.777 (95% CI: 0.727–0.820), whereas Asian studies demonstrated a pooled C-statistic of 0.845 (95% CI: 0.783–0.891). However, this difference was not statistically significant (*P* for interaction = 0.076), suggesting that model performance remains comparable across these geographic regions. Regarding validation types, studies utilizing internal validation yielded a pooled C-statistic of 0.813 (95% CI: 0.777–0.845), compared to 0.819 (95% CI: 0.557–0.942) for external validation; however, no statistically significant difference was observed between these groups (*P* for interaction = 0.956). Notably, the external validation subgroup displayed a markedly wide 95% CI, which can be attributed to the small number of contributing studies (*n* = 3) and the relatively modest sample sizes within individual external validation cohorts. Similarly, the analysis by publication language showed no significant disparity (*P* for interaction = 0.793), with pooled C-statistics of 0.818 (95% CI: 0.781–0.851) for English and 0.799 (95% CI: 0.618–0.907) for Chinese publications. The Chinese-language subgroup likewise exhibited a wide confidence interval, most likely resulting from the limited number of Chinese publications included and the smaller sample sizes of these studies. Finally, stratification by overall PROBAST risk of bias indicated that low-risk studies reported significantly better model performance (pooled C-statistic: 0.847, 95% CI: 0.807–0.879) than high-risk studies (pooled C-statistic: 0.762, 95% CI: 0.702–0.813), a difference that remained statistically significant (*P* for interaction < 0.05). Importantly, despite the wide confidence intervals observed in the external validation and Chinese-language subgroups, the subsequent leave-one-out sensitivity analysis (presented below) showed that sequentially excluding any single study produced only minimal fluctuations in the pooled C-statistic (range: 0.805–0.826), confirming the overall robustness of these subgroup findings.

**Figure 5:**
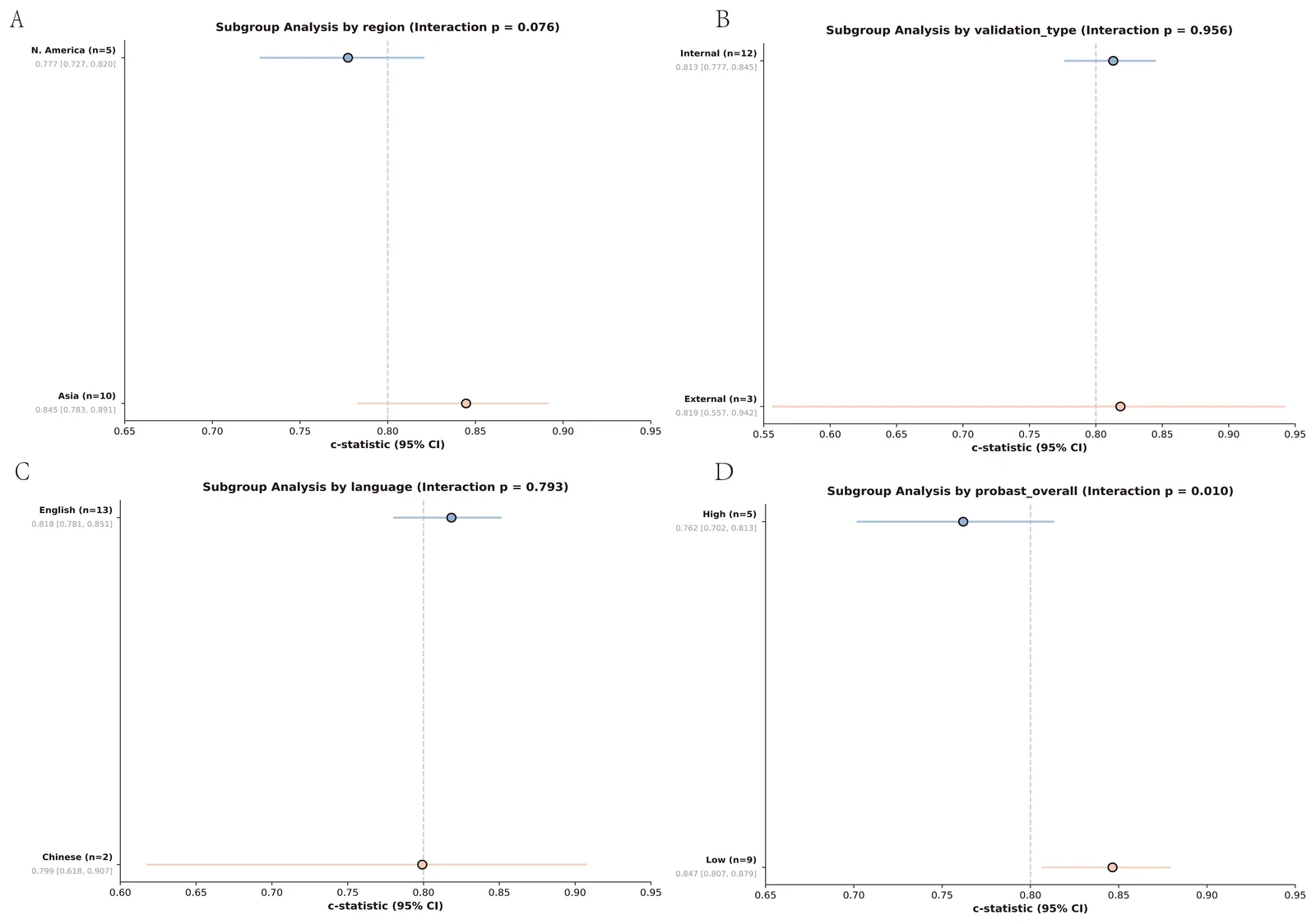
Subgroup analyses. Subgroup analyses of the pooled C-statistic stratified by **(A)** geographical region (North America vs. Asia), **(B)** validation type (internal vs. external), **(C)** publication language (English vs. Chinese), and **(D)** overall PROBAST risk of bias (low vs. high). Each subgroup is presented with the number of studies (*n*), pooled C-statistic estimate, and 95% CI. The vertical dashed line indicates the overall pooled effect (0.817). The interaction *P*-value tests for statistically significant differences between subgroups.

### Sensitivity analysis

The results of the sensitivity analysis are illustrated in **Figure 6**, demonstrating that after sequentially excluding each individual study, the pooled C-statistic fluctuated within a narrow range of 0.805 to 0.826. In the sensitivity analysis using the REML method with the Hartung–Knapp adjustment, the adjusted pooled C-statistic was 0.820 (95% CI: 0.773–0.858). These findings are highly consistent with our primary analysis based on the DL method (0.817, 95% CI: 0.781–0.847), demonstrating that the overall discriminative performance of the SA-AKI prediction models remains remarkably stable and robust despite high statistical heterogeneity. Notably, all recalculated estimates remained above 0.80, and their respective CIs consistently overlapped with the overall pooled effect size interval (0.781–0.847). Specifically, the lowest pooled C-statistic was observed following the exclusion of Su (2024) at 0.805 (95% CI: 0.769–0.836), while the exclusion of Peng (2023) yielded the highest value at 0.826 (95% CI: 0.791–0.856). Since no single study exerted a decisive influence on the aggregate effect size, we concluded that the findings of this meta-analysis possess high robustness.

**Figure 6:**
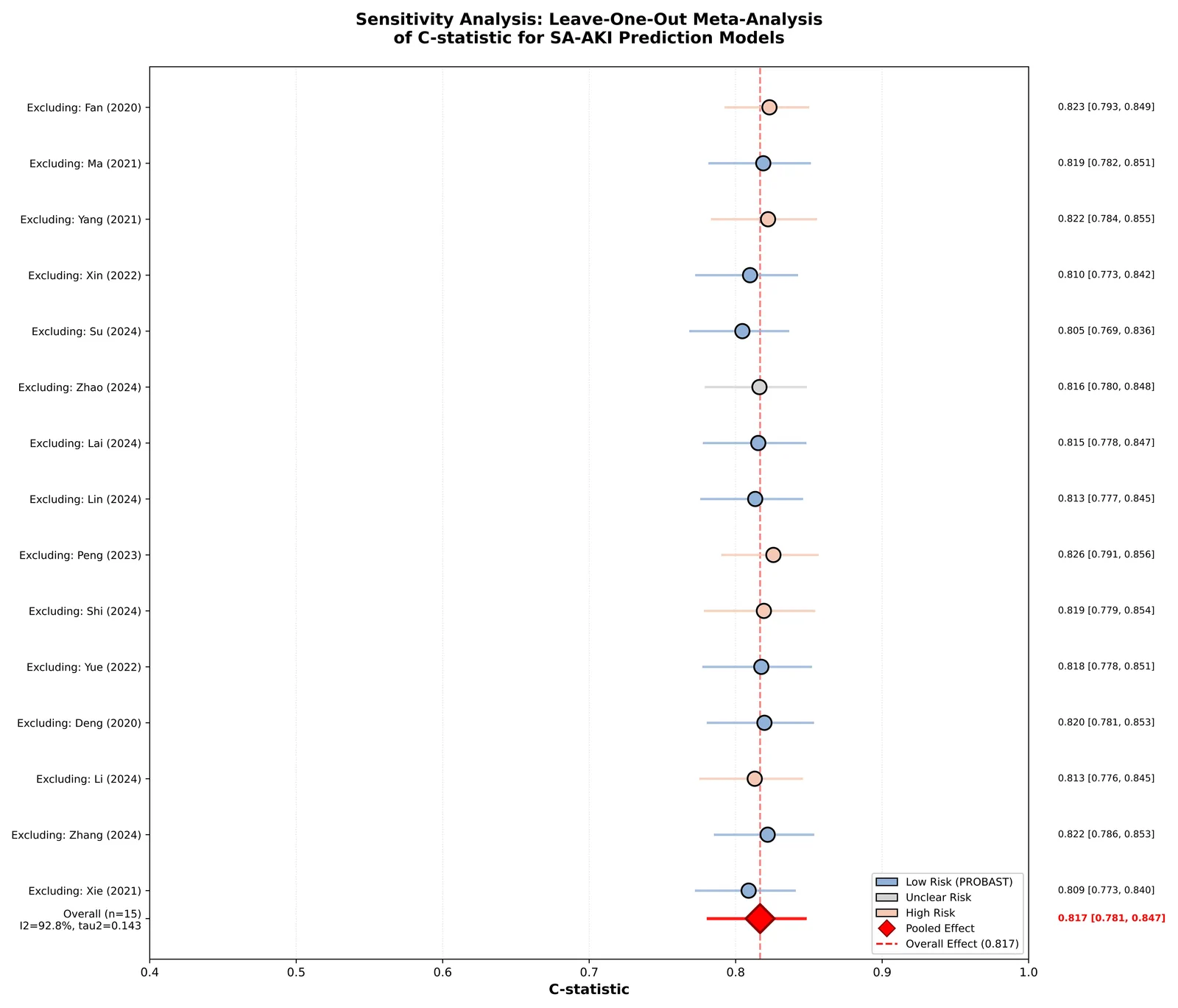
Sensitivity analysis: leave-one-out meta-analysis. Sensitivity analysis using the leave-one-out method, showing the pooled C-statistic after excluding each individual study. Each row represents the meta-analysis result when the specified study is omitted. Studies are color-coded by overall PROBAST risk of bias: blue for low risk, gray for unclear risk, and red for high risk. The vertical dashed red line indicates the overall pooled effect (0.817), and the diamond at the bottom represents the pooled estimate from the full analysis with all 15 studies included. The robustness of the meta-analysis is supported by the minimal fluctuation of pooled estimates (range: 0.805–0.826) across all leave-one-out scenarios.

## Discussion

This study presents a systematic review and meta-analysis of predictive models for SA-AKI. By synthesizing data from 15 studies involving a total of 46,490 patients, we demonstrated that existing models possess robust discriminatory power in identifying SA-AKI risk, as evidenced by a pooled C-statistic of 0.817 (95% CI: 0.781–0.847). This finding suggests that integrating clinical characteristics, laboratory markers, and comorbidities can provide clinicians with effective early-warning tools for decision-making. Compared to previous research focusing on AKI prediction in general hospitalized populations, our meta-analysis was specifically tailored to the septic cohort; notably, the superior discriminatory performance observed here reflects the critical role of sepsis-specific indicators—such as inflammatory response and organ dysfunction data—in enhancing predictive accuracy.

Despite the high overall discriminative power, the substantial statistical heterogeneity (*I*^2^ = 92.8%) observed across studies necessitates a cautious interpretation of these findings. Subgroup analysis suggested that geographic region might contribute to this variation; although studies conducted in Asia tended to show higher pooled C-statistics than those in North America, this difference did not reach statistical significance (*P* for interaction = 0.076). Specifically, the research by Fan et al. and Yang et al. (21, 29), based on the North American MIMIC-III database, yielded moderate C-statistics that were generally lower than those reported in multicenter or single-center studies from China, such as those by Su et al. or Zhang et al. (23, 35) Such numerical differences may stem from heterogeneity in healthcare systems regarding sepsis identification protocols, fluid resuscitation strategies, and the rigor of AKI diagnostic criteria implementation, rather than a true inherent superiority of region-specific models (32, 34).

The diversity of predictors was also identified as a core factor contributing to interstudy heterogeneity. Significant variations in variable selection were observed among the 15 included studies: Ma et al. substantially enhanced model performance by incorporating specific biomarkers like serum cystatin C (sCysC) and urinary NAG enzyme (28), while Deng et al. and Peng et al. focused on routine clinical data (e.g., fluid intake, creatinine, and duration of mechanical ventilation) within the first 24 h of ICU admission (25, 33). These contrasting approaches—”biomarker-oriented” versus “routine clinical data-oriented” model construction—resulted in variations as researchers captured different dimensions of the pathophysiological process of SA-AKI (36–38). Furthermore, Xie et al. emphasized the predictive value of antithrombin III (ATIII) in coagulation dysfunction-driven renal injury (22), whereas Xin et al. focused on the contribution of inflammatory markers such as interleukin-6 (IL-6) (30); this inconsistency in predictor dimensions further elucidates the origins of the observed heterogeneity.

From a methodological perspective, the impact of the risk of bias on model performance cannot be overlooked. According to the PROBAST assessment, the pooled performance of low-risk studies was significantly higher than that of high-risk studies, suggesting that deficiencies in certain studies—such as the handling of missing data, variable selection techniques (e.g., whether LASSO regression was applied for feature dimensionality reduction), or the assessment of calibration—may have led to biased results. In particular, the machine learning algorithms adopted by Yue et al. and Shi et al. (26, 27), while demonstrating outstanding discrimination, carry inherent risks of non-linearity and potential overfitting, making them statistically inconsistent with traditional logistic regression models (such as that of Yang et al. (29)) during synthesis. Furthermore, the online dynamic nomogram developed by Li et al. utilized a cross-sectional design, which differs fundamentally in time scale and outcome definition from the survival analysis models based on Cox regression used by Peng et al. (25, 34); these methodological design variations collectively constitute the primary sources of heterogeneity in this meta-analysis.

Nevertheless, some limitations should be noted. First, a direct comparison between traditional logistic regression and machine learning models was not analyzed in the subgroups, which might help clarify the distinct clinical roles of these two modeling types. Second, this analysis mainly evaluated model discrimination, as data on model calibration were unfortunately limited in the included literature. Finally, due to the small number of studies in the external validation subgroup, the resulting wide 95% confidence interval suggests that more data would be helpful to fully confirm the cross-cohort performance, and the methodological limitations of pooling C-statistics across highly heterogeneous contexts must be acknowledged. The included studies varied widely in machine learning algorithms, specific patient subpopulations, clinical settings, and the strictness of AKI criteria implementation. Therefore, the global pooled C-statistic should be interpreted as a macro-benchmark of the field rather than a direct tool for individual clinical settings. More importantly, C-statistics alone reflect only model discrimination and do not establish clinical usefulness or net benefit. However, it is critical to emphasize that a robust pooled C-statistic alone does not establish immediate bedside readiness for these models. Discrimination is only the first step in predictive modeling; clinical implementation demands rigorous evaluation of calibration, net benefit, and decision curve analysis (DCA) to determine clinical utility and define actionable thresholds for intervention. Furthermore, practical challenges such as the feasibility of real-time predictor collection and the transportability of algorithms across heterogeneous electronic health record (EHR) systems remain significant barriers. Prospective impact studies are fundamentally required to demonstrate that the deployment of these models can tangibly improve clinical decision-making and patient outcomes before widespread implementation can be recommended. Current SA-AKI literature critically lacks structured calibration data and DCA, which represents a vital gap that future research must address to translate predictive power into real-world clinical utility. Addressing these reporting gaps in future studies will further enhance the clinical guidance of these models.

Additionally, it is worth noting that traditional funnel plots and Egger’s test possess inherent limitations when applied to the pooled C-statistics of prediction models. Because the C-statistic is inherently bounded and heavily dependent on the case-mix and baseline risk of the study population, funnel asymmetry or distribution patterns can be driven by population heterogeneity rather than pure publication bias alone.

Regarding its clinical utility, although the point estimate for the external validation subgroup appeared acceptable, this finding must be interpreted with extreme caution. Due to the limited number of contributing studies and the resulting highly unstable confidence interval, the current evidence suggests that external validation of SA-AKI prediction models remains heavily limited and uncertain, rather than demonstrating true robustness. However, future research must continue to focus on model scalability (39–42). While this analysis confirms the effectiveness of existing tools, clinicians must consider the specific environment when selecting a model; for instance, the model designed by Lin et al. for the emergency department is better suited for early screening (32), whereas the machine learning model developed by Shi et al. for the ICU is more appropriate for data-intensive critical care settings (26). In conclusion, future efforts should be directed toward developing real-time prediction systems that incorporate dynamic physiological parameters and conducting large-scale, multicenter external validations across different regions. Such steps are essential to overcome the geographic limitations and statistical heterogeneity identified in current research, ultimately achieving precision protection of renal function in patients with sepsis.

The findings of this systematic review and meta-analysis hold profound implications for both future clinical applications and fundamental research. Clinically, by establishing a benchmark for model performance (pooled C-statistic: 0.817), our study provides a framework for selecting and implementing optimal early-warning tools tailored to specific care settings (e.g., emergency departments vs. intensive care units). It highlights the necessity for clinical systems to transition from static, traditional scoring to dynamic, automated, and population-specific prediction algorithms within electronic health records, which could drastically optimize personalized therapeutic strategies and window periods for renoprotection. Fundamentally, the high interstudy heterogeneity and distinct predictor dimensions unveiled in our analysis underscore the complex, multi-mechanistic nature of SA-AKI (ranging from inflammation to coagulation dysregulation). This mapping of predictive variables bridges the gap between clinical data and basic pathophysiology, offering clear directions for experimental researchers to explore novel multi-omics biological pathways. Future fundamental studies could utilize these identified key clinical phenotypes to guide the discovery of high-sensitivity biomarkers, ultimately advancing the paradigm of precision and translation medicine in critical care.

## Conclusion

Through a systematic review and meta-analysis, this study provided a comprehensive evaluation of the predictive performance and influential factors of existing models for SA-AKI. The results demonstrated that SA-AKI predictive models possess an overall moderate-to-good discriminatory power with a pooled C-statistic of 0.817. Although significant heterogeneity was observed across studies—suggesting that model performance is sensitive to various factors—the analysis revealed that studies with a low risk of bias reported significantly better performance than those with a high risk of bias. Conversely, study population, validation type, and publication language were found to have no statistically significant impact on the predictive performance of the models in the interaction test. However, the conclusion regarding validation type remains highly inconclusive and limited by the small number of external validation studies, leaving the true generalizability of these models uncertain.

These findings hold significant clinical and research implications. First, while the overall performance of existing models is acceptable, the substantial heterogeneity (*I*^2^ = 92.8%) and broad prediction intervals suggest that the current evidence base still possesses evident limitations. Second, the observed geographic disparities may reflect differences across regions in patient population characteristics, clinical practice patterns, data quality, or model development methodologies; this provides an important direction for the cross-regional validation and optimization of future models. Third, the significant association between risk of bias and model performance underscores the importance of rigorous methodological quality, suggesting that clinicians and policymakers should prioritize the selection and application of predictive models from strictly validated, low-risk-of-bias studies.

## Patient and public involvement

Patients and the public were not involved in the design, conduct, reporting, or dissemination plans of this research.

## Registration and protocol

This systematic review was prospectively registered with PROSPERO (ID: CRD420261347810) on 22 March 2026. The study was planned, conducted, and reported in adherence to this protocol. Minor protocol modifications regarding the intervention and study design fields were officially logged in the PROSPERO database during the study process to optimize clinical relevance, with no other deviations made to the eligibility criteria, primary outcomes, or statistical analysis plans.

## Reporting guidelines

This study was reported in accordance with the PRISMA 2020 statement.
